# Editorial: Pharmacogenetics and pharmacogenomics in psychiatry: challenges and opportunities

**DOI:** 10.3389/fphar.2026.1937839

**Published:** 2026-07-27

**Authors:** Janko Samardzic, Karel Allegaert, Kariofyllis Karamperis

**Affiliations:** 1 Institute of Pharmacology, Clinical Pharmacology and Toxicology, Medical Faculty, University of Belgrade, Belgrade, Serbia; 2 Department of Development and Regeneration, KU Leuven, Leuven, Belgium; 3 Department of Pharmaceutical and Pharmacological Sciences, KU Leuven, Leuven, Belgium; 4 Department of Hospital Pharmacy, Erasmus MC, Rotterdam, Netherlands; 5 Laboratory of Pharmacogenomics and Individualized Therapy, Department of Pharmacy, School of Health Sciences, University of Patras, Patras, Greece

**Keywords:** genetic markers, personalized medicine, pharmacogenetics, pharmacogenomics, psychiatry

## Introduction

Pharmacogenetics and pharmacogenomics play an increasingly important role across many areas of medicine and healthcare, offering the potential to improve treatment outcomes by tailoring therapies to individual genetic profiles. This is especially valuable in fields where treatment responses can vary widely between patients, and where a trial-and-error approach is still common (Mangalagliu et al., 2026; Brown et al., 2025). However, challenges remain in translating genetic insights into clinical practice. These include the complexity of genetic influences on disease and drug response, limited representation of diverse populations in genetic research, and concerns related to the ethical, legal, and regulatory aspects of genetic testing.

Despite these barriers, there are substantial opportunities to enhance treatment efficacy, reduce adverse drug reactions, and support the development of more precise, individualized therapies. Ongoing advances in genomic technologies, data analytics, and the integration of genetic data with other clinical and biological markers hold promise towards a more personalized, evidence-based approach to healthcare, also in the field of psychiatry (Sharew et al., 2024).

The implementation of testing on pharmacogenetics in routine care is still evolving. Significant hurdles include the need for validated genetic markers, clear clinical guidelines, and greater awareness, endorsement by agencies, and the need for education among healthcare providers. Large-scale, multi-ethnic studies are essential to identify reliable genetic predictors of treatment response and to ensure that personalized medicine benefits all populations equitably.

This Research Topic aims to explore the role of pharmacogenetics and pharmacogenomics in advancing personalized medicine in psychiatry, with a focus on improving drug efficacy, minimizing side effects, and enhancing individualized care across diverse healthcare settings. The Research Topic has 9 papers, offering a diverse range of perspectives, providing new data in two original (ancestry data, anxiety patterns in adolescents) and one case report (SIADH and QTc prolongation by escitalopram-quetiapine), providing 4 reviews on a diversity of pharmacogenetic topics, and very interesting, also 2 patient and clinicians perspectives papers on pharmacogenetic testing.

## Original papers and case reports

Chenchula et al. reported on the genetic polymorphisms in *CYP2D6* and *CYP2C19* in 509 adults living in central Indian, and hereby provided evidence of the high prevalence of actionable *CYP2D6* and *CYP2C19* variants in this region, since 86% of the participants had at least one clinically actionable polymorphism (Chenchula et al.). Interesting, and with a focus on 112 hospitalized adolescents (mean age 15 years, 101/112 females) experiencing a depressive episode, the added anxiolytics (typically alimemazine or hydroxyzine) were more associated with adverse drug reactions (vegetative/somatic, or mental), compared to *CYP2C19* genetic variants in the first 3 weeks of sertraline exposure (Ivashchenko et al.). This somewhat provides additional perspectives on the complexity and the multifactorial pattern of their pharmacotherapy. The case report is another illustration of this multifactorial pattern, describing a case report on severe SIADH and QTc prolongation induced by escitalopram-quetiapine interaction, in the setting of a CYP2C19 intermediate metabolizer at therapeutic doses. The authors hereby conclude that routine monitoring of electrolytes and electrocardiograms remains indispensable for patient safety, as pharmacogenetic screening and therapeutic drug monitoring may not effectively predict such idiosyncratic reactions in resource-constrained settings (Jiang et al.).

## Reviews

In a mini-review, the pharmacogenetics of risperidone in autism spectrum disorders in children were discussed. Genetic variation in the *CYP2D6* gene emerged as the most robust predictor of inter-patient variability in pharmacokinetics, while also pharmacodynamic polymorphisms emerged that were involved in dopaminergic, serotonergic and metabolic pathways, (Honório et al.). Blambila et al. reported in a focused review on *CYP2C19*, l *CYP2D6*, *SLC6A4* and *HTR2A* polymorphism as factors impacting the antidepressant response. Finally, Lock et al. reported on phenotype-related variation in pharmacogenomic effect sizes, and compared proximal (bioavailability, side effects, symptom severity) to distal (treatment) outcomes. They hereby consistently observed a large effect for proximal outcomes (Lock et al.). A final review focused on ancestry-related patters, synthesizing the evidence on pharmacogenetics relevant to psychotropics in Brazil. Interesting, all reviews called for polygenic models and the integration of more advanced techniques to guide individualized pharmacotherapy (Rodrigues et al.).

## Patient and clinician perspectives

Interesting, two papers reported on patient and clinician perspectives on pharmacogenetic testing and genome-guided treatment for pharmacotherapy related to psychiatric care. In a pilot study with semi-structured interviews and integrated in the GEMS (Genetics and Environment in Mental Health) study, the authors collected the perspectives of patients and clinicians on pharmacogenetics testing related to psychosis. Both were generally positive, with clinicians again stressing the multifactorial aspect. Access, understanding and logistics were perceived as barriers (Richards-Brown et al.). Koufaki et al. reported on 201 patients recruited in a prospective pharmacogenetics trial. These data showed that there is potential to enhance patient education and awareness about pharmacogenetics testing, while there was an overall positive patient attitude to pharmacogenetic-guided pharmacotherapy (Koufaki et al.).

## Discussion and conclusion

This Research Topic has limitations on its diversity of topics, but does include new data and information. The overarching theme is hereby the need to integrate pharmacogenetics as one of the covariates of multifactorial phenotype. It seems that pharmacogenetics and pharmacogenomics in psychiatry have matured and evolved from mechanistic promise to become potential actionable clinical tools, yet their full potential remains unrealized (Mangalagliu et al., 2026; Brown et al., 2025; Sharew et al., 2024). To take next steps, coordinated efforts are warranted to generate robust evidence, integrate pharmacogenetics into clinical workflows, and ensure equitable access cross populations, ancestries, and developmental stages.
